# Bone Health History in Breast Cancer Patients on Aromatase Inhibitors

**DOI:** 10.1371/journal.pone.0111477

**Published:** 2014-10-29

**Authors:** Marilyn L. Kwan, Joan C. Lo, Li Tang, Cecile A. Laurent, Janise M. Roh, Malini Chandra, Theresa E. Hahn, Chi-Chen Hong, Lara Sucheston-Campbell, Dawn L. Hershman, Charles P. Quesenberry, Christine B. Ambrosone, Lawrence H. Kushi, Song Yao

**Affiliations:** 1 Division of Research, Kaiser Permanente Northern California, Oakland, California, United States of America; 2 Department of Cancer Prevention and Control, Roswell Park Cancer Institute, Buffalo, New York, United States of America; 3 Department of Medicine and the Herbert Irving Comprehensive Cancer Center, Columbia University, New York, New York, United States of America; SUNY Upstate Medical University, United States of America

## Abstract

A cross-sectional study was performed to assess bone health history among aromatase inhibitor (AI) users before breast cancer (BC) diagnosis, which may impact fracture risk after AI therapy and choice of initial hormonal therapy. A total of 2,157 invasive BC patients initially treated with an AI were identified from a prospective cohort study at Kaiser Permanente Northern California (KPNC). Data on demographic and lifestyle factors were obtained from in-person interviews, and bone health history and clinical data from KPNC clinical databases. The prevalence of osteoporosis and fractures in postmenopausal AI users was assessed, compared with 325 postmenopausal TAM users. The associations of bone health history with demographic and lifestyle factors in AI users were also examined. Among all initial AI users, 11.2% had a prior history of osteoporosis, 16.3% had a prior history of any fracture, and 4.6% had a prior history of major fracture. Postmenopausal women who were taking TAM as their initial hormonal therapy had significantly higher prevalence of prior osteoporosis than postmenopausal AI users (21.5% vs. 11.8%, p<0.0001). Among initial AI users, the associations of history of osteoporosis and fracture in BC patients with demographic and lifestyle factors were, in general, consistent with those known in healthy older women. This study is one of the first to characterize AI users and risk factors for bone morbidity before BC diagnosis. In the future, this study will examine lifestyle, molecular, and genetic risk factors for AI-induced fractures.

## Introduction

Aromatase inhibitors (AI) have been replacing tamoxifen (TAM) as adjuvant hormonal therapy for postmenopausal women diagnosed with early stage, hormone receptor (HR)-positive breast cancer. The current third-generation AIs inhibit 96–99% *in vivo* aromatase enzyme activity [1], thereby decreasing endogenous estrogen levels far below levels from natural menopause [2]. This highly efficient estrogen depletion by AIs benefits breast cancer patients by extending recurrence-free survival superior to TAM [3], [4], [5]. However, AIs put patients at high risk of fractures due to the central role of estrogen in maintaining normal bone metabolism [6]. In contrast, TAM is generally believed to be bone-conserving [2]. Several expert groups have developed guidelines for evaluating fracture risk in breast cancer patients who are planning to start AI therapy [7], [8], [9], [10], so that the benefits and harms of AIs can be carefully assessed to make an educated decision on choice of hormonal therapy.

The aforementioned guidelines vary slightly but usually include bone mineral density (BMD) testing and clinical assessment of risk factors for fracture [7], [8], [9], [10]. Although BMD remains a strong predictor for fracture risk, several studies have shown that a large proportion of patients who experienced fragility fractures had T-scores in the non-osteoporotic range [11], [12], which highlights the importance of evaluation of BMD-independent risk factors, such as *a priori* bone health history, age, physical activity, smoking, and alcohol intake [13], [14]. However, to our knowledge, only a few small studies have examined bone health history among AI users prior to breast cancer diagnosis [15], [16], and no studies have investigated lifestyle factors and prior risk of fracture in this patient population. In a real-world clinical setting, it is unknown how bone health history would affect hormonal therapy choice among postmenopausal women with HR-positive breast cancer. These data will be important to inform treatment and prevention strategies for AI users as a high-risk population for bone morbidity. Furthermore, it is of clinical significance to also examine whether known risk factors for fractures are also relevant in breast cancer patients, considering the paradoxical role of estrogens in promoting carcinogenesis yet maintaining bone health.

In a cross-sectional analysis of one of the largest contemporary cohorts of breast cancer patients, we describe history of osteoporosis and fracture and the prevalence of risk factors for fracture (age, race/ethnicity, body mass index (BMI), physical activity, smoking, alcohol intake, and calcium and vitamin D supplement use) before breast cancer diagnosis among initial AI users. We also compare prevalence of bone health history in postmenopausal AI users with postmenopausal TAM users. Lastly, we examine associations of these fracture risk factors with prior history of osteoporosis and fracture outcomes in AI users.

## Materials and Methods

### Study Population

The Pathways Study is a prospective study of 4,505 women with newly diagnosed invasive breast cancer who are members of Kaiser Permanente Northern California (KPNC), a large, integrated health care delivery system covering the San Francisco-Oakland Bay Area, Sacramento, and surrounding counties. Recruitment was from January 2006 to April 2013 through rapid case ascertainment procedures designed to enroll women prior to initiation of chemotherapy, as described elsewhere [17]. Eligibility criteria included: KPNC female members at least 21 years of age; had no previous history of malignancy other than non-melanoma skin cancer; spoke English, Spanish, Cantonese, or Mandarin; and resided within a 65-mile radius of a field interviewer. The mean time from diagnosis to enrollment was 2.0 (±0.7) months.

For this bone health sub-study, women were included if they had at least one hormonal therapy prescription of an AI or TAM that was indicated for treatment of their first primary breast cancer. A total of 1,159 women who had no hormonal therapy, 27 women who initiated hormonal therapy after recurrence of their original breast cancer, and 4 women who initiated hormonal therapy after their second primary breast cancer were excluded. The final study population consisted of 3,315 eligible women. Based on complete hormonal therapy prescription data through December 2013, 2,157 (65.1%) were initial AI users, and 1,158 (34.9%) were initial TAM users. For this analysis of baseline bone health history, only the initial AI users were included. In selected analyses, 325 postmenopausal women who received TAM as their initial hormonal therapy drug were also included as a comparison group to postmenopausal AI users (n = 2,033).

### Clinicopathologic Characteristics

Clinical and diagnostic tumor characteristics were obtained from the KPNC Cancer Registry approximately four months post-diagnosis [18]. These included: stage at diagnosis, estrogen/progesterone receptor (ER/PR) positivity, HER2/neu (Her2) status, surgery type, and treatment received.

### Self-reported Participant Information

The baseline interview was conducted at enrollment into the cohort approximately two months post-diagnosis, and included interviewer and self-administered questionnaires on sociodemographics, diet, physical activity, smoking, established breast cancer risk factors, health history, and use of vitamin/mineral supplements. Anthropometric measures were also obtained at baseline.

Information was collected on hysterectomy and oophorectomy and associated dates of the surgery, and age or date of last period. Menopause was defined as the absence of menses for 12 consecutive months or more relative to the date of the baseline interview, or having a complete hysterectomy or oophorectomy of both ovaries.

Physical activity was assessed using an activity frequency questionnaire based on the validated Arizona Activity Frequency Questionnaire (AAFQ) [19]. Activities in four main domains were asked: household, recreational, transportation, and sedentary. Diet was assessed using a 139-item modified version of the Block 2005 food frequency questionnaire (FFQ) (NutritionQuest, Berkeley, CA). Alcohol consumption (beer, wine, and liquor), including frequency and portion size, was obtained on the FFQ.

### Pharmacy Data

Prescription drug data for nearly 100% of KPNC enrollees is recorded in the KPNC pharmacy database, including drug name, National Drug Code, dosage and therapeutic class; prescription dates and cost; dispensing and refills; and prescribing physician, thus minimizing recall bias [20]. The pharmacy database was accessed to identify any outpatient prescriptions of AIs (anastrozole, letrozole, and exemestane) and TAM after breast cancer diagnosis. Prescriptions of bisphosphonates (BP) any time before breast cancer diagnosis were also captured. BPs are inhibitors of bone resorption and commonly prescribed to treat osteoporosis and other related conditions.

### Prior Bone Outcomes

International Classification of Diseases, 9^th^ edition (ICD-9) outpatient and hospitalization diagnoses of bone outcomes were obtained from the KPNC electronic medical record (EMR). These diagnosis codes include: (1) osteoporosis (733.00–733.09); (2) any prior fracture involving the neck, trunk, upper and lower extremities (805, 807–815, 817–825, 827–829, excluding open fractures, fractures involving spinal cord injury, fractures of the face/skull, fingers and toes, and those associated with major trauma); and (3) any major osteoporotic fracture of the spine, humerus, wrist, or hip (805.0, 805.2, 805.4, 805.8, 812.0, 812.2, 813.4, 813.5, 820.0, 820.2, 820.8, excluding those associated with major trauma) were ascertained as previously described [21].

Considering potential under-diagnosis or documentation of osteoporosis by clinicians [22], [23], we assumed that if a woman was prescribed a BP prior to breast cancer diagnosis, and considering that BPs are usually indicated for clinical treatment of osteoporosis, she was most likely diagnosed with the condition. Therefore, we expanded our definition of osteoporosis to include any prescription of BP before breast cancer diagnosis regardless of whether or not an ICD-9 diagnosis code was present. Thus, osteoporosis was defined as having any relevant ICD-9 diagnosis code or any prior prescription of BP.

### Statistical Analysis

Analyses of initial AI or TAM use in postmenopausal breast cancer patients by select characteristics, including prior history of osteoporosis and fracture, were conducted using logistic regression with adjustment for age at breast cancer diagnosis as a continuous variable.

In the overall initial AI user group, we calculated odds ratios (OR) and 95% confidence intervals (CI) using logistic regression to estimate the associations of lifestyle and clinical factors at breast cancer diagnosis with prior history of 1) osteoporosis, 2) any fracture, and 3) any major osteoporotic fracture. All models were adjusted for age, race/ethnicity, menopausal status, and year of breast cancer diagnosis, and all p-values were two-tailed with a significance level of 0.05. Analyses were repeated after excluding those diagnosed with breast cancer before menopause. The results were similar to those from the overall initial AI user group and thus are not presented here.

All analyses were conducted in SAS version 9.3 (Cary, N.C.).

### Ethics Statement

The study was approved by the KPNC institutional review board.

## Results

As shown in **Table 1**, the mean age at breast cancer diagnosis among AI users was 64.4 years, with 2,033 (94.5%) initially diagnosed after menopause and 118 (5.5%) before menopause. As expected, the majority of the initial AI users were diagnosed with early stage disease (AJCC stage I–III) (98.4%). The study cohort was multi-ethnic, with 71.4% White, 10.4% Hispanic, 10.3% Asian, 5.8% African American, and 2.1% other race/ethnicity. The mean BMI at baseline was 29.0 kg/m^2^, with 31.8% being overweight and 38.2% obese. The median (IQR) of moderate-vigorous physical activity at baseline was 19.5 (7.3–39.1) metabolic equivalent (MET)-hours/week. Over half of the patients were never smokers at baseline (52.0%), 42.5% were former smokers, and only 5.5% were current smokers. Alcohol intake was light at baseline, with 27.9% being never drinkers, and among those who drank, the median intake was 3.10 grams/day. Nearly two-thirds of the patients did not take either calcium or vitamin D supplements at baseline (63.2%), 16.5% took calcium, 12.6% took vitamin D, and 7.7% took both supplements.

**Table 1:** Baseline characteristics of study cohort by initial use of aromatase inhibitor (AI) or tamoxifen (TAM).

			Overall	Postmenopausal Only		
			AI (Initial Use)	AI (Initial Use)	TAM (Initial Use)	p value^1^
			n = 2157	n = 2033	n = 325	
			n (%)	n (%)	n (%)	
**Age at Breast Cancer (BC) Diagnosis (years)**						**<0.0001**
<50			80 (3.7)	28 (1.4)	47 (14.5)	
50–59			640 (29.7)	581 (28.6)	101 (31.1)	
60–69			891 (41.3)	883 (43.4)	99 (30.5)	
≥70			546 (25.3)	541 (26.6)	78 (24.0)	
Mean (SD)			64.4 (9.1)	65.2 (8.5)	62.0 (11.2)	
**Menopausal Status**						–
Premenopausal			118 (5.5)	0 (0)	0 (0)	
Postmenopausal			2033 (94.5)	2033 (100)	325 (100)	
**AJCC Stage at BC Diagnosis**						**0.0003**
I			1165 (54.0)	1120 (55.1)	218 (67.1)	
II			751 (34.8)	693 (34.1)	85 (26.2)	
III			206 (9.6)	187 (9.2)	20 (6.2)	
IV			35 (1.6)	33 (1.6)	2 (0.6)	
**Race/Ethnicity**						0.62
White			1541 (71.4)	1467 (72.2)	222 (68.3)	
African American			124 (5.8)	115 (5.7)	22 (6.8)	
Asian			221 (10.3)	202 (9.9)	31 (9.5)	
Hispanic			225 (10.4)	205 (10.1)	43 (13.2)	
Other			46 (2.1)	44 (2.2)	7 (2.2)	
**BMI (kg/m** 2 **)**						**<0.0001**
<25			642 (30.0)	595 (29.5)	127 (39.4)	
25–29.9			680 (31.8)	645 (31.9)	108 (33.5)	
≥30			816 (38.2)	780 (38.6)	87 (27.0)	
Mean (SD)			29.0 (6.5)	29.0 (6.5)	27.6 (6.4)	
**Mod-Vig Physical Activity (MET-hours/week)**						
Mean (SD)			27.6 (28.6)	27.6 (28.9)	33.0 (34.0)	**0.03**
Median (IQR)			19.5 (7.3–39.1)	19.4 (7.1–39.1)	24.4 (9.3–45.4)	
**Smoking History**						0.28
Never			1118 (52.0)	1046 (51.5)	183 (56.3)	
Former			913 (42.5)	874 (43.0)	129 (39.7)	
Current			118 (5.5)	111 (5.5)	13 (4.0)	
**Alcohol Intake (g/day)**						0.24
Never			511 (27.9)	477 (27.5)	81 (29.7)	
≤median^2^			665 (36.2)	618 (35.7)	106 (38.8)	
>median			659 (35.9)	637 (36.8)	86 (31.5)	
**Vitamin Supplement Use**					**0.003**	
None			1263 (62.7)	177 (55.0)		
Calcium			334 (16.6)	61 (18.9)		
Vitamin D			259 (12.9)	43 (13.4)		
Both			158 (7.9)	41 (12.7)		
		**<0.0001**				
No			1792 (88.2)	225 (78.5)		
≤6			165 (8.1)	50 (12.3)		
>6			76 (3.7)	30 (9.2)		
**Any Fracture Before BC Diagnosis (years)**					0.27	
No			1695 (83.4)	272 (83.7)		
≤6			179 (8.8)	23 (7.1)		
>6			159 (7.8)	30 (9.2)		
		0.13				
No			1937 (95.3)	306 (94.2)		
≤6			60 (3.0)	11 (3.4)		
>6			36 (1.8)	8 (2.5)		

NOTE: Pharmacy data through December 31, 2013; Missing data for entire cohort: menopausal status (n = 9), BMI (n = 28), smoking (n = 13), alcohol (n = 552), vitamin supplements (n = 37).

1 Logistic regression adjusted for age at breast cancer diagnosis as a continuous variable.

2 Median (overall) = 3.10 g/day, median (postmenopausal women) = 2.90 g/day.

3 Osteoporosis defined by ICD-9 code (733.00–733.09) or any prior bisphosphonate prescription.

4 Major fracture includes fracture of spine, humerus, wrist, or hip.

Among the initial AI users, 11.2% had a prior history of osteoporosis, including 3.5% at 6 years or more before breast cancer diagnosis, and 7.7% within 6 years (**Table 1**). 16.3% of the patients had a prior history of any fracture, including 7.6% at 6 years or more before cancer diagnosis, and 8.7% within 6 years. For major fractures of the spine, humerus, wrist, or hip, 4.6% had a prior history of these fractures, including 1.7% at 6 years or more before cancer diagnosis, and 2.9% within 6 years.

Among postmenopausal patients with HR-positive breast cancer, the majority received AIs as their initial hormonal therapy (n = 2,033, 86.2%), and 325 patients (n = 13.8%) received TAM as their initial hormonal therapy. No apparent secular trend was found in the use of AIs relative to TAM during the study period (2005–2013). To explore whether bone health history or other known risk factors for fractures might affect the choice of hormonal therapy drugs, comparisons were conducted between postmenopausal initial AI users and postmenopausal initial TAM users. As shown among the postmenopausal women in **Table 1**, compared to initial AI users, women on TAM were younger (mean age 62.0 years vs. 65.2 years, p<0.0001), more likely to have stage I disease (67.1% vs. 55.1%, p = 0.0003), less obese (mean BMI 27.6 kg/m^2^ vs. 29.0 kg/m^2^, p = 0.0001), and more physically active (median 19.4 MET-hours/week vs. 24.4 MET-hours/week, p = 0.03). No differences in race/ethnicity, smoking history, or alcohol intake were found between the two groups. For bone health history prior to breast cancer diagnosis, although initial TAM users were younger, they had a significantly higher prevalence of prior osteoporosis than initial AI users (21.5% vs. 11.8%, p<0.0001), but a similar prevalence of any prior fracture (16.3% vs. 16.6%, p = 0.27) and major prior fracture (5.9% vs. 4.8%, p = 0.13). Consistent with a higher prevalence of prior osteoporosis compared with AI users, TAM users were more likely to take calcium and/or vitamin D than AI users (45.0% vs. 37.3%, p = 0.003).

In Tables 2–4, the associations of selected risk factors with prior history of osteoporosis and fractures among overall initial AI users are presented. In models adjusted for age, race/ethnicity, menopausal status, and year of breast cancer diagnosis, older age (60–69 y OR = 2.43; 95% CI: 1.58, 3.72; ≥70 y OR = 5.65; 95% CI: 3.68, 8.69; p for trend<0.0001) and being Asian (OR = 1.82; 95% CI: 1.17, 2.81) were associated with higher odds of prior osteoporosis, whereas increasing BMI (overweight OR = 0.55; 95% CI: 0.40, 0.76; obese OR = 0.32; 95% CI: 0.22, 0.45; p for trend<0.0001) and being African American (OR = 0.38; 95% CI: 0.15, 0.94) were associated with lower odds (**Table 2**).

**Table 2:** Baseline characteristics in relation to osteoporosis^1^ before breast cancer diagnosis (BC) in aromatase inhibitor (AI) users.

	Osteoporosis BeforeBC Diagnosis – Yes		Osteoporosis BeforeBC Diagnosis – No	OR^2^	95% CI^2^	p for trend
	n = 242		n = 1915			
	n (%)		n (%)			
**Age at BC Diagnosis (years)**						**<0.0001**
<60		31 (12.8)	689 (36.0)	Ref		
60–69		95 (39.3)	796 (41.6)	**2.43**	**(1.58, 3.72)**	
≥70		116 (47.9)	430 (22.5)	**5.65**	**(3.68, 8.69)**	
**Race/Ethnicity**						–
White		182 (75.2)	1359 (71.0)	Ref		
African American		5 (2.1)	119 (6.2)	**0.38**	**(0.15, 0.94)**	
Asian		30 (12.4)	191 (10.0)	**1.82**	**(1.17, 2.81)**	
Hispanic		19 (7.9)	206 (10.8)	0.86	(0.52, 1.43)	
Other		6 (2.5)	40 (2.1)	1.46	(0.60, 3.54)	
**BMI (kg/m** 2 **)**						**<0.0001**
<25		114 (47.3)	528 (27.8)	Ref		
25–29.9		77 (32.0)	603 (31.8)	**0.55**	**(0.40, 0.76)**	
≥30		50 (20.8)	766 (40.4)	**0.32**	**(0.22, 0.45)**	
**Mod-Vig Physical Activity (MET-hours/week)**						–
Never	16 (6.7)		92 (4.8)	Ref		
≤median^3^	102 (42.5)		915 (48.2)	0.92	(0.51, 1.67)	
>median	122 (50.8)		893 (47.0)	1.25	(0.69, 2.27)	
**Smoking History**						–
Never	134 (55.4)		984 (51.6)	Ref		
Former	101 (41.7)		812 (42.6)	0.86	(0.65, 1.15)	
Current	7 (2.9)		111 (5.8)	0.62	(0.28, 1.39)	
**Alcohol Intake (g/day)**						–
Never	77 (34.8)		434 (26.9)	Ref		
≤median^3^	73 (33.0)		591 (36.6)	0.73	(0.51, 1.05)	
>median	71 (32.1)		589 (36.5)	0.72	(0.50, 1.04)	
**Vitamin Supplement Use**						–
None	146 (60.8)		1202 (63.5)	Ref		
Calcium	40 (16.7)		311 (16.4)	1.08	(0.73, 1.58)	
Vitamin D	34 (14.2)		235 (12.4)	1.06	(0.69, 1.63)	
Both	20 (8.3)		145 (7.7)	1.03	(0.61, 1.74)	

NOTE: Pharmacy data through December 31, 2013.

1 Osteoporosis defined by ICD-9 code (733.00–733.09) or any prior bisphosphonate prescription.

2 Logistic regression adjusted for age, race/ethnicity, menopausal status, and year of breast cancer diagnosis.

3 Median (physical activity) = 20.9 metabolic equivalent (MET)-hours/week; median (alcohol intake) = 3.1 g/day.

**Table 3:** Baseline characteristics in relation to any fracture before breast cancer (BC) diagnosis in aromatase inhibitor (AI) users.

	Any Fracture BeforeBC Diagnosis – Yes		Any fracture BeforeBC Diagnosis – No	OR^1^	95% CI^1^	p for trend
	n = 352		n = 1805			
	n (%)		n (%)			
**Age at BC Diagnosis (years)**						**<0.0001**
<60	70 (19.9)		650 (36.0)	Ref		
60–69	143 (40.6)		748 (41.4)	**1.76**	**(1.27, 2.43)**	
≥70	139 (39.5)		407 (22.6)	**3.13**	**(2.24, 4.37)**	
**Race/Ethnicity**						–
White	268 (76.1)		1273 (70.5)	Ref		
African American	24 (6.8)		100 (5.5)	1.30	(0.81, 2.08)	
Asian	17 (4.8)		204 (11.3)	**0.50**	**(0.30, 0.84)**	
Hispanic	34 (9.7)		191 (10.6)	0.97	(0.66, 1.44)	
Other	9 (2.6)		37 (2.1)	1.37	(0.65, 2.90)	
**BMI (kg/m** 2 **)**						0.86
<25	93 (26.6)		549 (30.7)	Ref		
25–29.9	135 (38.6)		545 (30.5)	**1.43**	**(1.06, 1.92)**	
≥30	122 (34.9)		694 (38.8)	1.00	(0.74, 1.36)	
**Mod-Vig Physical Activity (MET-hours/week)**						–
Never	26 (7.5)		82 (4.6)	Ref		
≤median^2^	164 (47.0)		853 (47.6)	0.83	(0.51, 1.35)	
>median	159 (45.6)		856 (47.8)	0.84	(0.51, 1.37)	
**Smoking History**						–
Never	178 (50.7)		940 (52.3)	Ref		
Former	152 (43.3)		761 (42.3)	0.93	(0.73, 1.19)	
Current	21 (6.0)		97 (5.4)	1.24	(0.75, 2.07)	
**Alcohol Intake (g/day)**						–
Never	89 (30.4)		422 (27.4)	Ref		
≤median^2^	88 (30.0)		576 (37.4)	0.73	(0.52, 1.01)	
>median	116 (39.6)		544 (35.3)	1.00	(0.73, 1.38)	
**Vitamin Supplement Use**						–
None	203 (58.8)		1145 (64.0)	Ref		
Calcium	60 (17.4)		291 (16.3)	1.21	(0.88, 1.68)	
Vitamin D	49 (14.2)		220 (12.3)	1.12	(0.78, 1.61)	
Both	33 (9.6)		132 (7.4)	1.21	(0.79, 1.86)	
**Osteoporosis Before BC Diagnosis**						–
No	267 (75.9)		1648 (91.3)	Ref		
Yes	85 (24.2)		157 (8.7)	**2.86**	**(2.10, 3.89)**	

NOTE: Pharmacy data through December 31, 2013.

1 Logistic regression adjusted for age, race/ethnicity, menopausal status, and year of breast cancer diagnosis.

2 Median (physical activity) = 20.9 metabolic equivalent (MET)-hours/week; median (alcohol intake) = 3.1 g/day.

**Table 4:** Baseline characteristics in relation to major fracture^1^ before breast cancer (BC) diagnosis in aromatase inhibitor (AI) users.

		Major Fracture Before BC Diagnosis – Yes		Major Fracture Before BC Diagnosis – No	OR^2^	95% CI^2^	p for trend
		n = 99		n = 2058			
		n (%)		n (%)			
**Age at BC Diagnosis (years)**							**<0.0001**
<60		16 (16.2)		704 (34.2)	Ref		
60–69		32 (32.3)		859 (41.7)	1.54	(0.81, 2.93)	
≥70		51 (51.5)		495 (24.1)	**4.23**	**(2.29, 7.83)**	
**Race/Ethnicity**							–
White		82 (82.8)		1459 (70.9)	Ref		
African American		3 (3.0)		121 (5.9)	0.52	(0.16, 1.70)	
Asian		4 (4.0)		217 (10.5)	0.47	(0.17, 1.30)	
Hispanic		6 (6.1)		219 (10.6)	0.59	(0.25, 1.37)	
Other		4 (4.0)		42 (2.0)	2.20	(0.76, 6.39)	
**BMI (kg/m** 2 **)**							0.38
<25		28 (28.3)		614 (30.1)	Ref		
25–29.9		39 (39.4)		641 (31.4)	1.33	(0.80, 2.21)	
≥30		32 (32.3)		784 (38.5)	0.93	(0.55, 1.59)	
**Mod-Vig Physical Activity (MET-hours/week)**							–
Never	15 (15.5)			93 (4.6)	Ref		
≤median^3^	40 (41.2)			977 (47.8)	**0.33**	**(0.17, 0.64)**	
>median	42 (43.3)			973 (47.6)	**0.36**	**(0.18, 0.70)**	
**Smoking History**							–
Never	49 (49.5)			1069 (52.2)	Ref		
Former	48 (48.5)			865 (42.2)	1.05	(0.69, 1.60)	
Current	2 (2.0)			116 (5.7)	0.44	(0.11, 1.86)	
**Alcohol Intake (g/day)**							–
Never	24 (28.2)			487 (27.8)	Ref		
≤median^3^	24 (28.2)			640 (36.6)	0.76	(0.43, 1.38)	
>median	37 (43.5)			623 (35.6)	1.16	(0.67, 2.00)	
**Vitamin Supplement Use**							–
None	61 (63.5)			1287 (63.2)	Ref		
Calcium	13 (13.5)			338 (16.6)	0.82	(0.44, 1.53)	
Vitamin D	12 (12.5)			257 (12.6)	0.89	(0.46, 1.72)	
Both	10 (10.4)			155 (7.6)	1.20	(0.59, 2.45)	
**Osteoporosis Before BC Diagnosis**							–
No			67 (67.7)	1848 (89.8)	Ref		
Yes			32 (32.3)	210 (10.2)	**3.15**	**(1.98, 5.02)**	

NOTE: Pharmacy data through December 31, 2013.

1 Major fracture includes fracture of spine, humerus, wrist, or hip.

2 Logistic regression adjusted for age, race/ethnicity, menopausal status, and year of breast cancer diagnosis.

3 Median (physical activity) = 20.9 metabolic equivalent (MET)-hours/week; median (alcohol intake) = 3.1 g/day.

Associations of patient characteristics and lifestyle factors with prior history of any fracture are given in **Table 3**. Older age was associated with higher odds of any prior fracture (p for trend<0.0001), whereas being Asian (OR = 0.50; 95% CI: 0.30, 0.84) was associated with lower odds of any prior fracture. In contrast to associations with prior osteoporosis, no significant increasing BMI trend was found, yet being overweight was associated with increased odds of any prior fracture (OR = 1.43; 95% CI: 1.06, 1.92). Finally, as expected, prior history of osteoporosis was associated with increased odds of prior fracture (OR = 2.86; 95% CI: 2.10, 3.89). Associations of patient characteristics and lifestyle factors with prior major fracture were largely consistent with any prior fracture, but with wider CIs (**Table 4**). Unique to analyses of major fracture, however, any moderate-vigorous physical activity was associated with reduced odds of major fracture in the AI users (≤median 20.9 MET-hours/week OR = 0.33 95% CI: 0.17, 0.64; >median OR = 0.36; 95% CI: 0.18, 0.70).

## Discussion

In a large contemporary cohort of breast cancer survivors who were initially treated with AIs, we found that 11.2% had a prior history of osteoporosis, 16.3% any fracture, and 4.6% major fracture before breast cancer diagnosis. Although the majority of postmenopausal women were initially treated with AIs, a sizable proportion (13.8%) was initially treated with TAM. Furthermore, these TAM users had nearly twice the prevalence of prior osteoporosis compared with initial AI users. Finally, the associations of selected risk factors with prior history of bone health outcomes in breast cancer patients initially treated with AIs were largely consistent with those expected from the healthy older population [13], [14].

To our knowledge, our observational study is the largest to date to describe the use of AIs as primary hormonal therapy in conjunction with prior bone morbidity. A previous study of 343 women with early-stage breast cancer about to initiate AI therapy reported 22.2% with osteoporosis and 11.4% with any fracture [16]. Another study of 497 breast cancer patients also at the onset of AI therapy found 19.1% with non-vertebral fractures [15]. Compared with our prevalence findings of 11.2% osteoporosis and 16.3% any fracture, Servitja et al. reported a higher rate of osteoporosis but a lower rate of fracture, whereas Bouvard et al. reported a higher rate of fracture. While these studies were limited by small sample size, they did collect baseline bone health measures of BMD, spinal X-rays, and 25-hydroxyvitamin D [25-(OH)D] concentrations. Our current analysis does not consider these data, yet in future prospective analyses of fracture risk, we will be incorporating BMD measures and 25(OH)D concentrations around baseline entry into the cohort.

For postmenopausal women diagnosed with early stage, HR-positive breast cancer, AIs have been shown to have superior efficacy in lowering risk of recurrence compared with TAM, and thus have become the preferable choice for this patient subgroup. However, TAM remains a viable choice for initial hormonal therapy for those who seek to avoid AIs’ musculoskeletal effects. This is likely true for women deemed to have low risk of recurrence but are susceptible to fractures, as suggested by our results. In postmenopausal patients in our study, initial TAM users were slightly younger than initial AI users, yet the former had significantly higher prevalence of osteoporosis history. However, initial TAM users were more likely to have stage I disease than initial AI users, suggesting their risk of recurrence was lower. A lower risk of recurrence coupled with a higher risk of fracture might have influenced physicians and patients to favor TAM over AIs as their first choice of initial hormonal therapy. This speculation was further strengthened by the findings of higher usage of calcium and/or vitamin D supplement and higher physical activity in the initial TAM users than in the initial AI users. As supplement use and physical activity were surveyed soon after breast cancer diagnosis, we could not assess whether these data represent exposure status before or after the diagnosis of osteoporosis. Higher usage of supplements and being more physically active might have been in response to being diagnosed with osteoporosis (reverse causality). It is also interesting to note that a small proportion of initial AI users were diagnosed with breast cancer before menopause. Most likely those patients experienced menopause due to chemotherapy or radiation therapy and were subsequently eligible for AI therapy.

Among initial AI users, we identified several risk factors associated with history of osteoporosis, including older age, Asian race, and lower BMI. Older age was also associated with fracture history and being physically active was associated with lower risk of major prior fracture. These associations were in the same direction as expected in a general healthy older population, suggesting common mechanisms for osteoporosis and fracture regardless of later breast cancer diagnosis. Although smoking and alcohol consumption are risk factors for osteoporosis and fracture in non-cancer patients, we did not find such associations in initial AI users, possibly due to the small proportion (5.5%) of current smokers in the study and light alcohol intake in the cohort (median intake of 3.10 grams/day among drinkers). We also found that Asian AI users had a higher risk of osteoporosis but lower risk of prior fracture than Whites. This observation potentially reflects known racial/ethnic differences of lower bone mineral density, yet decreased fracture risk, in healthy Asians compared with Whites [24], [25], [26].

When comparing our proportion of prior fracture to healthy postmenopausal women at a similar age in the Women’s Health Initiative (WHI) [27], initial postmenopausal AI users were approximately two times less likely to have a history of fracture (16.6% vs. 34.4%, p = 0.0039). The lower rate of prevalent osteoporosis and fracture seen among breast cancer patients in our study may be explained by the paradoxical association between high BMD and breast cancer risk [28]. As estrogen plays a central role in bone growth and maintenance, it also drives the development of breast cancer. Therefore, at the time of diagnosis, women with breast cancer may have had higher lifetime estrogen exposure and associated BMD than non-cancer women at a similar age, which may explain the lower prevalence of osteoporosis and fracture among breast cancer patients seen in our study.

Our study was based on a large prospective breast cancer cohort from one of the nation’s largest integrated healthcare delivery systems where complete electronic medical records and pharmacy information are available on all patients. This enabled us to accurately assess bone health history through their health insurance membership with Kaiser Permanente. Moreover, the cohort was established in 2005, a time concurrent with the widespread use of AIs. Our study was further strengthened by the availability of extensive demographic and lifestyle information collected at the time of diagnosis through in-person interviews.

Nevertheless, our study is not without limitations. Due to its cross-sectional nature, we could not infer causality of the selected risk factors for prevalent osteoporosis and fracture among breast cancer patients. In addition, osteoporosis was potentially under-diagnosed in our patient population. However, this is not a unique challenge to this study. Clinicians vary in their use and coding of the term osteoporosis such that it may be identified after BMD testing, after a primary care visit, at the time of a specialty visit, at the time of bisphosphonate initiation, or at the time of fracture. Further, a diagnosis may not always mean that the T-score is in the osteoporosis range, and a diagnosis could also be made in the presence of a fragility fracture while BMD is only in the osteopenic range. There have also been temporal changes in the frequency of osteoporosis diagnosis [29], which may explain the greater proportion of AI users with *a priori* osteoporosis diagnosis, as AIs did not become the preferential choice of hormonal therapy for postmenopausal breast cancer patients until the mid-2000’s. To overcome this limitation, we classified patients with BP treatment but without an ICD-9 diagnosis of osteoporosis as osteoporotic. The results were similar when we relied only on osteoporosis ICD-9 diagnosis. Furthermore, the prevalence of prior spine fractures could have been underestimated due to asymptomatic fractures not being coded by the physician, but for our analyses, we considered spine fractures as clinically diagnosed. Finally, given our access to electronic medical records on the cohort, we estimate that only 35% had BMD measurements before breast cancer diagnosis based on Current Procedural Terminology (CPT) codes, thus precluding us from classifying osteoporosis status based on BMD data for the entire cohort. To note, the prevalence of prior BMD screening in our cohort is greater than 16% reported in a small study of BMD screening adherence among 342 breast cancer patients on AIs, and these women were also members of an integrated health care system [30].

To conclude, a small proportion of breast cancer patients initially treated with AIs had a positive history of osteoporosis or fracture before their cancer diagnosis. A history of osteoporosis might influence the choice of initial hormonal therapy drug among patients with low risk of recurrence. Risk factors for osteoporosis and fracture before breast cancer diagnosis were similar to those among healthy older women. Given that adverse effects on bone health is a common comorbidity concern for many cancer survivors [31], our findings may have wide clinical applicability beyond breast cancer that emphasize the importance of full consideration of prior bone health history before initiation of cancer treatment.
